# Generation of simulated data for Bengali text localization in natural images

**DOI:** 10.1016/j.dib.2023.109568

**Published:** 2023-09-14

**Authors:** Sourav Saha, Md. Easin Arafat, Md Aminul Haque Palash, Dewan Md Farid, M. Shamim Kaiser

**Affiliations:** a Department of Computer Science and Engineering, United International University, United City, Madani Avenue, Badda, Dhaka 1212, Bangladesh; b Institute of Information Technology, Jahangirnagar University, Savar, Dhaka 1342, Bangladesh; c Department of Research and Development, Pioneer Alpha, Dhaka 1205, Bangladesh; d Department of Computer Science and Engineering, Chittagong University of Engineering & Technology, Rawjan, Chittagong 4349, Bangladesh

**Keywords:** Text editing, Text style transfer, Simulated image, Bengali language, Generative adversarial network

## Abstract

In the domain of vision-based applications, the importance of text cannot be underestimated due to its natural capacity to provide accurate and comprehensive information. The application of scene text editing systems enables the modification and enhancement of textual material included in natural images while maintaining the integrity of the overall visual layout. The complexity of keeping the original background context and font styles when altering, however, is an extremely difficult challenge considering the changed image must perfectly blend with the original without being altered. This article contains significant simulated data on the dynamic features of digital image editing, advertising, content development, and related fields. The system comprises key components such as 2D simulated text on the styled image $\left(i_{s}\right)$, text image $\left(i_{t}\right)$, masking of text $\left(mask_{t}\right)$, real background image $\left(t_{b}\right)$, real sample image $\left(t_{f}\right)$, text skeleton $\left(t_{sk}\right)$, and text styled image $\left(t_{t}\right)$. The source dataset contains diverse components such as background images, color variations, fonts, and text content, while the synthetic dataset consists of 49,000 randomly generated images. The dataset provides both researchers and practitioners with a rich resource for identifying and evaluating these dynamic features. The dataset is publicly accessible via the link: https://data.mendeley.com/datasets/h9kry9y46s/3

Specifications Table

**Table:** 

Subject	Computer Vision and Pattern Recognition

Specific subject area	Image processing, Advertising Image Editing, Text Style Transfer, Typography
Data format	Simulated digital images (.png)
Type of data	Raw
Data collection	The background images utilized in this data collection process have been collected from a diverse range of resources. Additionally, a specific set of Bengali fonts and the corresponding text and their colors were chosen for the purpose of generating our simulated data.
Data source location	Dhaka, Bangladesh
Data accessibility	Repository name: Mendeley Data Data identification number: 10.17632/h9kry9y46s.2 Direct URL to data: https://data.mendeley.com/datasets/h9kry9y46s/3 Instructions for accessing these data: https://github.com/srv-sh/BSTE-dataset

## 1 Value of the Data

• This dataset can be used to train deep learning models for a variety of applications, including digital image modification, advertising, and content creation.
• This dataset can help deep learning researchers interested in text editing systems and alterations. This dataset will primarily help them to generate scene text editing style transfer systems, notably in the context of the Bengali language.
• The dataset consists of two portions, with the source dataset consisting of background images, color, fonts, and text, and the synthetic dataset containing 49,000 images selected at random. The expansion of this dataset will increase the accuracy of deep learning models' kinds of data [1].
• This dataset is applicable to numerous applications, including interpretable modules, text style transfer, and text editing networks in Bengali scene text editing.
• Identifying any pattern of texts that can be used to change the text instances in a text image without destroying its realistic appearance. This dataset has no limit so standalone text editing tools can be made for the Bengali language. Moreover, advanced lightweight applications that use this dataset could help digital ads and image editing to make a better typography system [2].

## 2 Data Description

The text serves as a fundamental medium for the repository and transmission of information, playing a crucial role in modern times where various forms of communication depend extensively on textual cues and labels. Image/video text, also known as simple text or scene text, incorporates textual content present in both dynamic visual media and static images. In various vision-based applications, scene text includes a wide range of semantic information that is valuable and pertinent to the task. In numerous cases, scene text includes a wide range of meaningful information that is useful and relevant to the task. Automation in image searching [3], quick translation [4], and robot movements [5] are some examples.

Aside from that, this article focuses on two subsets of datasets: source datasets and synthetic datasets. We scale the text image in our experiment to a height of 64 pixels while keeping the original aspect ratio while producing synthetic data. In addition, examples of synthetic data depicting various image types in a left-to-right order are included in machine learning and deep learning models for the purpose of accurate text style transfer. Tables 1 and 2 show descriptions of the source and synthetic datasets, respectively.

**Table 1:** Description of the source dataset.

**Table 2:** Description of the synthetic dataset.

Synthetic Data	Amount of Data	Description
$i_{s}$ –> styled image	7000	Refers to the placement of textual content over a selected styled image, enhancing visual appeal and conveying information in a combined visual format.
$i_{t}$ –> text image	7000	A visual representation of text converted into an image format is often used to preserve formatting, font styles, and artistic aspects of the text for various design and communication purposes
$mask_{t}$ –> masking of text	7000	The technique of selectively revealing or concealing specific parts of text using masks, enabling creative effects or precise control over text visibility within a design or composition.
$t_{b}$ –> real background image	7000	The process of adjusting the dimensions of a real background image to fit within specified size constraints, ensuring compatibility with various display platforms and maintaining visual integrity.
$t_{f}$ –> real sample image	7000	Involves overlaying textual content onto a pre-existing real sample image, and combining visual elements for enhanced communication or aesthetic purposes.
$t_{sk}$ –> text skeleton	7000	An outline or simplified representation of the structure of textual characters, typically used for initial layout planning, font design, and as a basis for creative text manipulation.
$t_{t}$ –> text styled image	7000	An image wherein the text is not only present but has been meticulously styled using various typography elements such as fonts, sizes, colors, and formatting, resulting in a visually appealing and communicative design.

## 3 Experimental Design, Materials and Methods

The dataset preparation consists of two steps: data collection and method to simulate the data. This section briefly describes each of these steps to prepare a Simulated image dataset.

### 3.1 Data collection

**Fig. 1:**
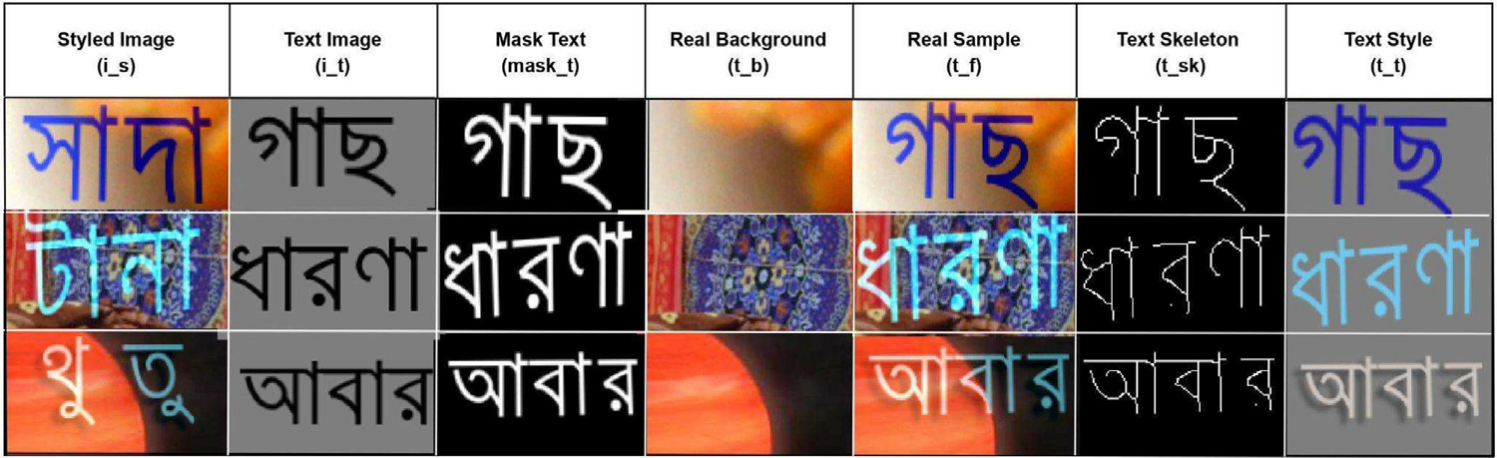
Examples of synthetic data depicting various image types, arranged in a left-to-right direction. The images include styled images, text images, real background text images, ground truth images, text skeleton images, and text style images.

In this article, we have compiled a background image dataset from [6], capturing essential backgrounds, genuine text, and text structures through image skeletonization, guided by ground truth in Fig. 1. The dataset contains paired styled images $\left(i_{s}\right)$ and text images $\left(i_{t}\right)$. The original images act as genuine backgrounds $\left(t_{b}\right)$ for target text images, while text stroke segmentation masks $\left(mask_{t}\right)$ are provided to segment it in the target text images. Ground truth images $\left(t_{f}\right)$ serve as reference outcomes. Furthermore, text skeletons $\left(t_{sk}\right)$ outline text structures in target images. Text style $\left(t_{t}\right)$ combines background, text, and stroke segmentation for synthesized images. This dataset supports research in text recognition, image synthesis, and text-to-image algorithms.

### 3.2 Method follows to simulate the data

**Fig. 2:**
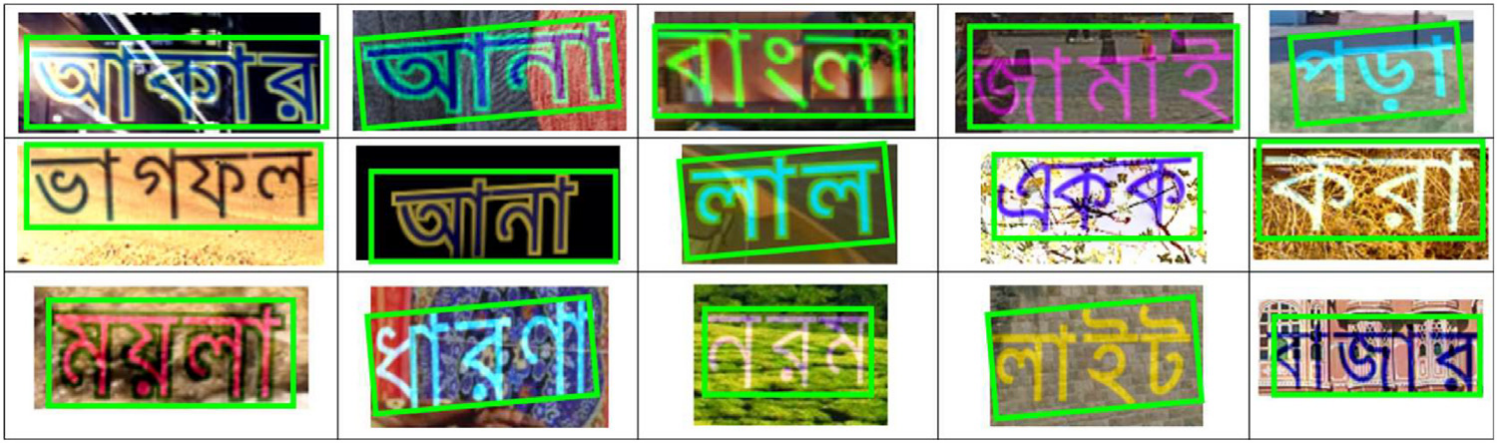
Simulated text-localization image samples.

In generating simulated synthetic data, we resize text images to a height of 64 pixels while preserving the original aspect ratio. Fig. 2 illustrates a collection of sample images depicting simulated text localization.

To simulate this, please visit our GitHub repository which provides concise instructions on how to reproduce all the things step-by-step that commence by creating a dedicated Conda environment named 'your_dataset' using the specified Python version 3.9.0. Activate this environment using the command 'conda activate your_dataset'. Subsequently, ensure the installation of the requisite packages by executing 'pip install -r requirements.txt'.

**Fig. 3:**
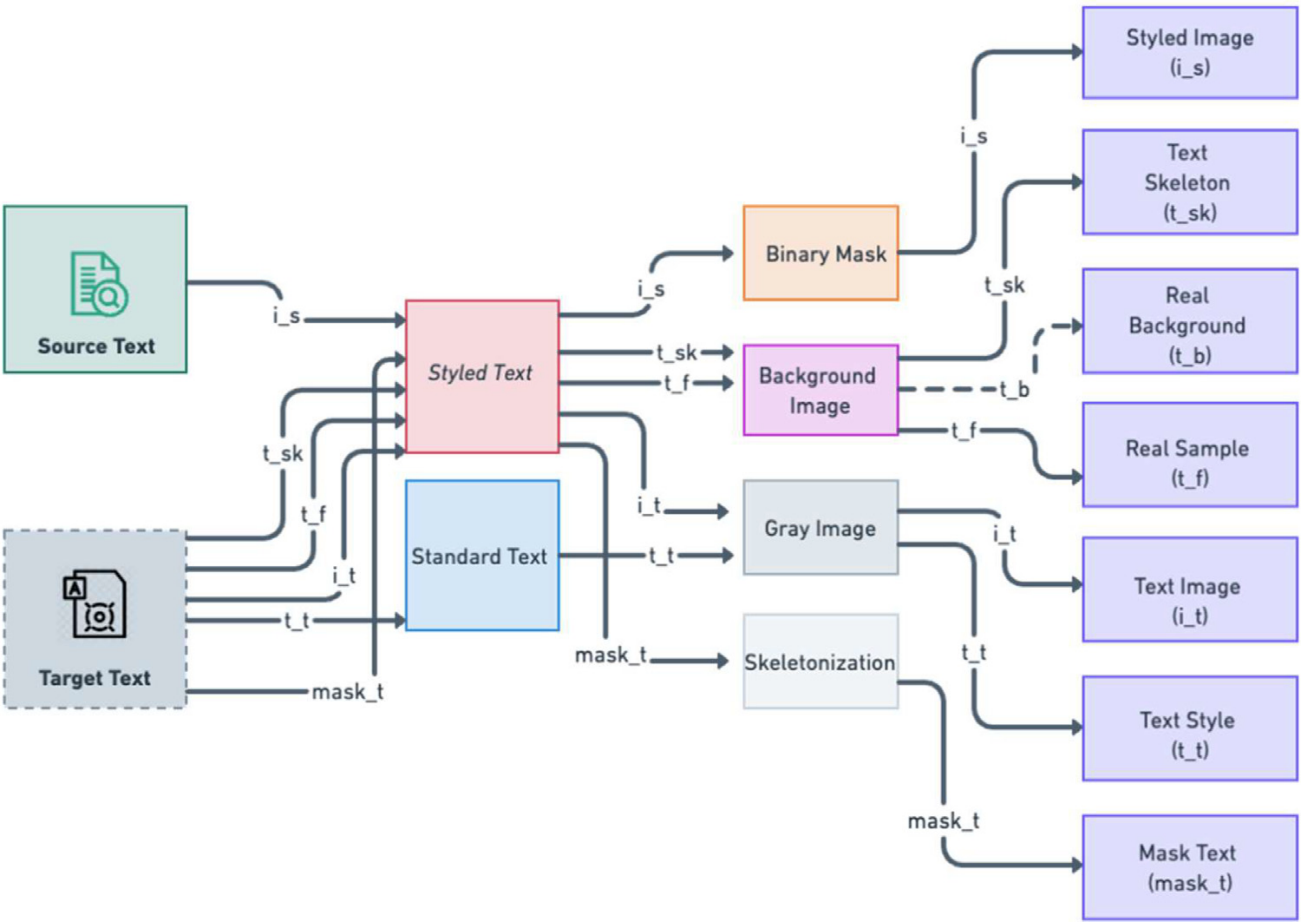
The visual representation of the essential stages in synthetic data generation. Starting with source and target text definition, followed by model creation, simulation, and data refinement using techniques like binary masks, background images, grayscale conversions, and skeletonization, culminating in labeled data for supervised learning. This approach supports the development and training of machine learning models.

Fig. 3 visually illustrates the sequential stages of synthetic data production. Our network architecture, based on pix2pix [7], operates with an Adam optimizer [8] using β1=0.5 and β2=0.999. Training persists until output stability is observed. The learning rate begins at $2\times 10^{-4},$ later decreasing to $2\times 10^{-6}\;$after 30 epochs. Both generator and discriminator undergo spectral normalization [9], with the generator also employing batch normalization [10]. The batch size is 8, and input images are resized to approximately w×64, maintaining the aspect ratio.

For data generation, adherence to specific file and folder arrangements is crucial. Commence by downloading and extracting the contents of the “Source Dataset.zip” file from the Mendeley data link. Establish a folder labelled `utils' at the root directory of the repository. Within this 'utils' directory, extract the `text.zip' file, retaining only the 'text' folder. Similarly, extract the 'fonts.zip' file, isolating the 'fonts' folder. Transfer the extracted contents of the `background.zip' file to the 'background' folder within 'utils'. Similarly, populate the 'colors' folder within `utils' with the contents of the 'color.zip' file. Place the `colors.cp' file within the 'utils' directory. Finally, execute the 'datagen.py' script to initiate the data generation process. This comprehensive approach ensures the successful reproduction of the data generation workflow.

## Limitations

We have plans to further enhance our text editing resources more useful by adding support for more than one language pair. With this growth, we want to reach a more diverse and global audience and make sure that our tools for editing text can be used well in different language settings. These measures will look at not only how well the language is used, but also how easy it is to read, how well it makes sense, and how well it fits the situation. By doing this, we hope to give practitioners a deeper and more detailed view of how well our simulated dataset work.

## Ethics Statement

There are no studies involving human participants done by any of the authors in this article. The datasets used in the article are open to the public. For the usage of these datasets, proper citation rules should be maintained.

## CRediT Author Statement

**Sourav Saha:** Conceptualization, Methodology, Formal analysis, Writing – original draft. **Md. Easin Arafat:** Validation, Resources, Data curation, Visualization, Writing – original draft. **Md Aminul Haque Palash:** Data Generation, Validation, Writing – original draft. **Dewan Md Farid:** Formal analysis, Validation. **M. Shamim Kaiser:** Analytical reviewing, Validation, Supervision.

## Data Availability

Simulated data for bangali text localisation in natural image (Original data) (Mendeley Data).
